# A hypoxia response element in the *Vegfa* promoter is required for basal *Vegfa* expression in skin and for optimal granulation tissue formation during wound healing in mice

**DOI:** 10.1371/journal.pone.0180586

**Published:** 2017-07-07

**Authors:** Domenic Ciarlillo, Christophe Celeste, Peter Carmeliet, Derek Boerboom, Christine Theoret

**Affiliations:** 1 Département de biomédecine vétérinaire, Faculté de médecine vétérinaire, Université de Montréal, St-Hyacinthe, Québec, Canada; 2 Laboratory of Angiogenesis and Vascular Metabolism, Vesalius Research Center, University of Leuven, Leuven, Belgium; University of Pisa, ITALY

## Abstract

Hypoxia in skin wounds is thought to contribute to healing through the induction of hypoxia inducible factor-1 (HIF-1). Although HIF-1 can regulate the expression of vascular endothelial growth factor A *(Vegfa)*, whether hypoxia and HIF-1 are required to induce *Vegfa* expression in the context of wound healing is unknown. To test this hypothesis, we evaluated *Vegfa* expression and wound healing in mutant mice that lack a functional HIF-1 binding site in the *Vegfa* promoter. Full-thickness excisional wounds were made using a biopsy punch, left to heal by second intention, and granulation tissue isolated on a time course during healing. mRNA levels of *Vegfa* and its target genes platelet-derived growth factors B *(Pdgfb)* and stromal cell-derived factor-1 *(Sdf1)* were measured by RT-qPCR, and HIF-1alpha and VEGFA protein levels measured by immunoblotting. Lower levels of *Vegfa*, *Pdgf1* and *Sdf1* mRNA were found in intact skin of mutant mice relative to wild-type controls (n = 6 mice/genotype), whereas levels in granulation tissue during wound healing were unaltered. VEGFA protein levels were also lower in intact skin of the mutant versus the wild-type mice. Decreased *Vegfa* mRNA levels in skin of mutant mice could not be attributed to decreased HIF-1alpha protein expression, and were therefore a consequence of the loss of HIF-1 responsiveness of the *Vegfa* promoter. Comparative histologic analyses of healing wounds in mutant and wild-type mice (n = 8 mice/genotype) revealed significant defects in granulation tissue in the mutant mice, both in terms of quantity and capillary density, although epithelialization and healing rates were unaltered. We conclude that HIF-1 is not a major regulator of *Vegfa* expression during wound healing; rather, it serves to maintain basal levels of expression of *Vegfa* and its target genes in intact skin, which are required for optimal granulation tissue formation in response to wounding.

## Introduction

Hypoxia within skin wounds arises primarily in response to traumatic destruction of the cutaneous vasculature and augmentation of cellular oxygen consumption rates due to temporary increases in cellular density and metabolic activity [1–4]. Acute hypoxia favors wound healing [1, 2] because low oxygen tension levels promote angiogenesis [5], fibroplasia [1, 2, 4, 5], epithelialization [1, 4], and extracellular matrix (ECM) synthesis [1, 4]. These effects are regulated, at least in part, by hypoxia-induced increases in the expression of various growth factors including transforming growth factor (TGF)-ß1 [1, 2, 4], platelet-derived growth factor (PDGF) [4] and vascular endothelial growth factor (VEGFA) [2, 4].

Hypoxia triggers the accumulation of hypoxia-inducible factor (HIF)-1, the cellular hypoxia sensor, which is the key element in the process of oxygen homeostasis and in the re-establishment of blood vessels in hypoxic areas [1, 4]. HIF-1 is a heterodimeric transcription factor that induces the expression of a plethora of genes that mediate adaptive responses to hypoxia such as angiogenesis [1, 4, 6] and cell proliferation/survival [4]. It exerts its transcriptional regulatory effects by binding a site named the hypoxia-response element (HRE) found within the promoters of various genes including *VEGFA*, angiopoietin (*ANGPT)1*, *ANGPT2*, *PDGFB*, glucose transporter (*GLUT)-1* and stromal cell-derived factor (*SDF)-1* [3, 6–8].

Angiogenesis is a physiological process whereby new blood vessels form from pre-existing vessels [9, 10]. In the wound environment, these new vessels are required to deliver essential nutrients and oxygen to mitotically active cells, thereby ensuring that healing progresses normally [11]. VEGFA is a key regulator of angiogenesis in a variety of developmental and physiological processes including wound healing [12, 13]. VEGFA is also capable of upregulating other factors, such as PDGFB and SDF-1, which play an important role in angiogenesis [14–16]. HIF-1 is thought to be a major regulator of *Vegfa* expression in several cell types, and acts via a well-characterized HRE in the *Vegfa* promoter [6, 17]. However, whether HIF-1 acts to induce *Vegfa* expression in the context of cutaneous wound healing has not been established. In the current study, we tested this idea *in vivo* using the *Vegfa*^∂/∂^ mouse model, which lacks a functional HRE in the *Vegfa* promoter, and therefore cannot respond to hypoxia/HIF-1 with an increase in *Vegfa* transcriptional activity [18]. We predicted that *Vegfa* upregulation in response to wounding would fail to occur in mutant mice, and that wound healing would be compromised due to poor angiogenesis and the presence of granulation tissue of inferior quantity and/or quality. While our experimental goal was achieved, the outcome of our study demonstrated that, contrary to prediction, HIF-1 is required for basal expression of *Vegfa* in intact skin, rather than in granulation tissue during wound healing.

## Materials and methods

### Mice

*Vegfa*^tm2Pec^ (hereafter *Vegfa*^∂/∂^) mice were as originally described [19]. Briefly, the *Vegfa*^∂/∂^ strain was provided to us on a mixed background, containing FVB, C57/Bl(6) and likely other strains as well, with variable coat colors. *Vegfa*^∂/∂^ and wild-type mice were generated by backcrossing Vegfa^∂/+^ mice; consequently, the *Vegfa*^∂/∂^ experimental animals were the littermates of the wild-type controls.

Wild type and mutant mice used for all experimentation were male and ranged from 6–8 weeks in age. All mice were healthy upon commencement of experimentation. Animals were housed individually under standardized conditions with controlled temperature, humidity, and a 12-hour-day/12-hour-night light cycle. Animals had free access to water and standard mouse chow. This study was conducted at the Faculté de médecine vétérinaire in strict accordance with the guidelines for the care and use of laboratory animals, as sanctioned by the Canadian Council on Animal Care. The protocol was approved by the Comité d’éthique de l’utilisation des animaux de l’Université de Montréal (permit number: Rech-1635). All surgery was performed under anesthesia, and all efforts were made to minimize suffering.

### Part I: Molecular profile

For the first part of the study, 6 *Vegfa*^∂/∂^ and 6 wild type mice were used. On day 0, mice were anesthetized by isoflurane inhalation 20 minutes after subcutaneous administration of Metacam™ (meloxicam, 4mg/kg–Boehringer Ingelheim, St. Joseph, Missouri, USA). The dorsum was clipped and prepared aseptically. Three full-thickness excisional wounds were made to below the level of the *panniculus carnosus*, one on each side of midline and a third one centrally located just caudal to the other two wound sites, using a sterile, disposable, 6 mm-diameter biopsy punch and scissors; the excised tissue (intact skin) was kept as a day 0 sample. Wounds were left uncovered to heal by secondary intention.

Post-operatively, mice were injected subcutaneously with 1 ml of warm, 0.9% sterile saline solution for fluid replacement. All mice were placed on a warming pad until mobile and were then returned to their cage (cardboard paper bases were used as substrate) where they were further warmed until fully awake. Mice received a subcutaneous dose of Metacam™ (4mg/kg, SID) on postoperative days 1 and 2.

Animals were anesthetized as per wound creation surgery on days 5, 7 and 9; at each of these times, a single wound was randomly selected for harvest. On day 9 the animals were euthanized by carbon dioxide (CO_2_) inhalation after wound harvest. Wound sampling consisted of creating a new 6 mm-diameter wound encompassing the initial wound to ensure that the entire region of interest was collected. Mice received a subcutaneous dose of Metacam™ (4mg/kg, SID) on the day of tissue collection. Metacam™ (4mg/kg, SID) was also administered for 2 consecutive days following each sampling procedure. Tissue samples were embedded in Optimal Cutting Temperature compound (OCT—Tissue-Tek, Torrance, CA, USA), snap-frozen in liquid nitrogen and then stored at -80°C.

The wellbeing of all experimental mice was assessed once to twice daily. Post-operative general health was evaluated primarily through behavioural modifications; appetite, agitation, and activity levels were some of the parameters used to assess the presence of pain. Altered facial expressions (such as orbital tightening) were also taken into consideration. As previously stated, mice received a subcutaneous dose of Metacam™ (4mg/kg, SID) on the day of surgery and for 2 consecutive days following all surgical interventions. Additional post-operative analgesia was not necessary for any of the experimental subjects.

#### Real-time RT-PCR

Wound tissue samples were thawed and the peripheral and deeper wound sections were discarded, keeping only granulation tissue. Conversely, the totality of day 0 samples (i.e., intact skin) was kept. Total RNA was isolated and purified using the RNeasy Fibrous Tissue Mini kit (Qiagen Sciences, Maryland, USA), following the manufacturer’s instructions. Synthesis of cDNA was done using a SuperScript VILO cDNA synthesis kit (Thermo Fisher Scientific, Life Technologies, Carlsbad, CA, USA) following the manufacturer’s instructions. Quantitative polymerase chain reaction (qPCR) was carried out in a Bio-Rad CFX96 Touch Real Time PCR Detection System, using the SSoAdvanced Universal SYBR Green Supermix (Bio-Rad Laboratories, Hercules, CA, USA), following the manufacturer’s instructions. Custom oligonucleotide primers were designed via Life Technologies, Inc (Table 1) and standard curves were generated by serial dilution of a preparation of total cDNA. Expression levels of genes were calculated relative to the housekeeping gene Ribosomal Protein-L19 (*Rpl19)* [20].

**Table 1:** Primers used for real-time qPCR.

Gene	Forward 5’– 3’	Reverse 5’– 3’
***Vegfa***	GGAGACTCTTCGAGGAGCACTT	GGCGATTTAGCAGCAGATATAAGAA
***Pdgfb***	GAGGGGGATCCCATTCCTGA	GCCCCATCTTCATCTACGGA
***Sdf-1***	TTCTTCGAGAGCCACATCGC	TCAGCCGTGCAACAATCTGA

#### Western blot

Intact skin and granulation tissue samples were weighed then placed in extraction buffer (Tissue Protein Extraction Reagent [T-PER^®^ –Thermo Scientific, Rockford, IL, USA)] supplemented with a protease inhibitor tablet [Complete—Sigma-Aldrich, Mannheim, Germany]) as per manufacturers’ instructions, and homogenized using a PowerGen 125 (Fisher Scientific) tissue homogenizer. The solution was then centrifuged at 10,000 xg for 5 minutes at 14^°^C. The protein concentration of the supernatants was measured using the Bradford protein assay. Twenty microgram (μg) protein samples were separated on a 12% agarose gel then transferred to a polyvinylidene fluoride (PVDF) membrane (Immobilon-P Transfer Membranes—EMD Millipore Corporation, Billerica, M, USA). Membranes were blocked in 5% milk for 1 hour at room temperature and then incubated overnight in a 5% albumin solution containing the primary antibody at a dilution of 1:500. The primary antibodies used to detect HIF-1α and VEGFA were obtained from Santa Cruz Biotechnology (sc-53546 and sc-507, respectively). The following day, the membranes were incubated for 60 minutes in a 12.5% milk solution containing horseradish peroxidase (HRP)-conjugated secondary antibody, and detection of immunoreactive proteins was performed with a chemiluminescent HRP substrate (Immobilon–Millipore) and the Chemidoc™ MP imaging system (Bio-Rad Laboratories, Inc.). Beta-actin (ACTB) (β-actin—sc-47778; dilution 1:500,000; Santa Cruz Biotechnology) was used as a loading control. Signal intensity measurements were obtained using Image Lab™ software version 5.2.1 (Bio-Rad Laboratories, Inc. Hercules, CA, USA).

### Part II: Wound healing assay

Eight mutant (*Vegfa*^δ/δ^) and 8 wild type mice were used for the second part of the study. The same anesthetic, surgical and analgesic protocols applied in part I of experimentation were followed in part II, except two wounds were created on either side of midline using a sterile, disposable 10 mm-diameter biopsy punch and a scalpel blade. The excised tissue (intact skin) was kept as a day 0 sample. Wounds were photographed (baseline measurement) and were left uncovered to heal by secondary intention.

Mice were immobilized through the use of general anesthesia and each wound was photographed using a digital camera alongside a scale bar, on postoperative days 3, 7, 10 and 14. The wound surface area was calculated in pixels, by a blinded observer, using ImageJ software (http://imagej.nih.gov/ij/), and expressed as a percentage of the original wound surface area to determine the rate of healing. On day 7 the right-sided wounds were collected from each animal using an 8 mm-diameter biopsy punch and scalpel blade following photography. The analgesic protocol outlined in part I was also applied to the second experiment. On day 14, the animals were euthanized by CO_2_ inhalation then the left-sided wounds were collected. All samples were harvested with a biopsy punch to include the remaining wound and the surrounding skin, placed flat on a piece of filter paper, *subcutis* side down, then divided in half. One half was fixed in 10% neutral-buffered formalin solution for 24h then stored in 70% alcohol at 4^°^C until processing in paraffin wax for histology. The second half was embedded in OCT then snap-frozen in liquid nitrogen and stored at -80°C until real-time (RT)-qPCR and Western Blot assays.

#### Histological analyses

**Fig 1:**
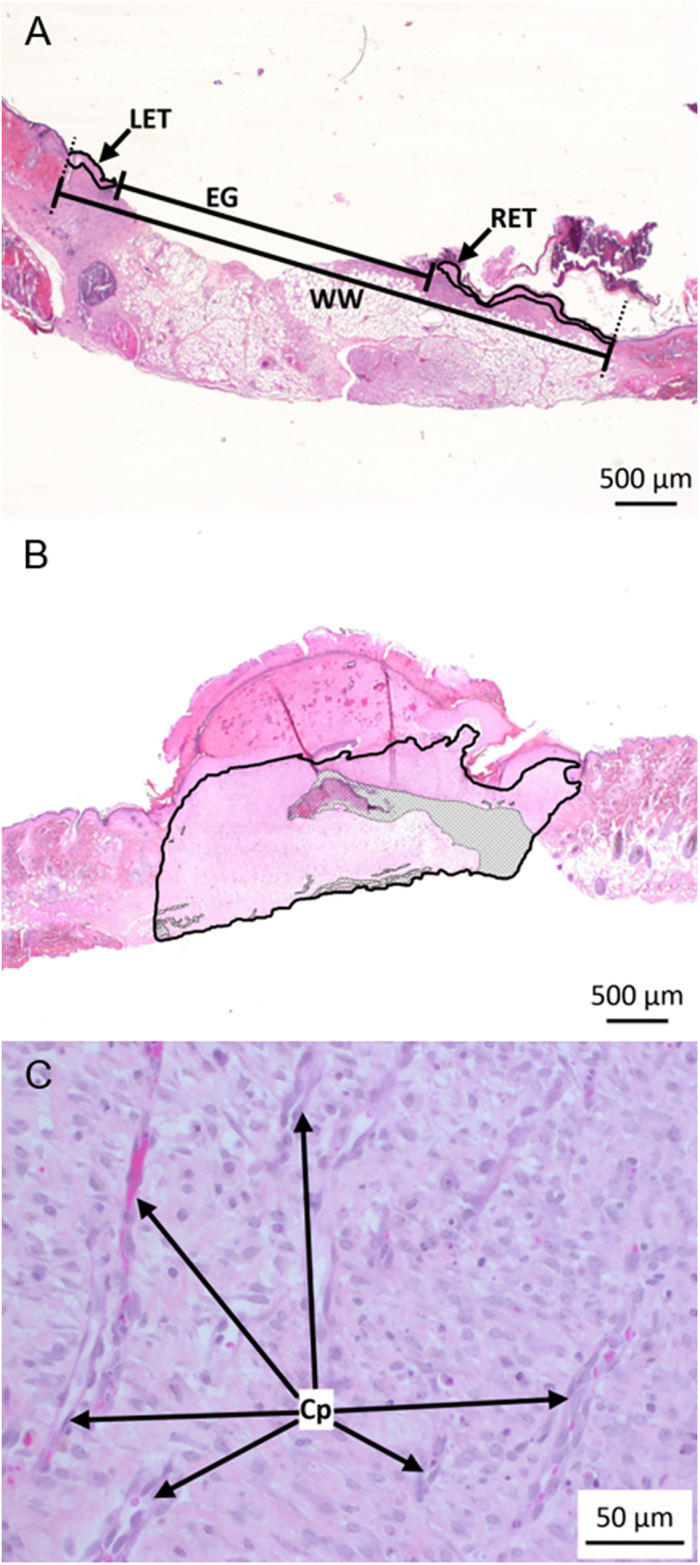
Measurement of neo-epidermal coverage, area of granulation tissue, and capillary density, in healing wounds of wild type and mutant (*Vegfa*^∂/∂^) mice. (A) Representative photomicrograph of a 7 day-old wound in a wild type mouse. Measurements of the left (LET) and right epidermal tongues (RET), epidermal gap (EG) and wound width (WW) were used to evaluate the progress of wound healing. (B) Representative photomicrograph of a 7 day-old wound in a wild type mouse with the granulation tissue circumscribed. Adipose tissue or muscle found within the demarcated zone (grey hatched area) was excluded from the surface area measurement. (C) Representative photomicrograph showing examples of new capillaries (Cp) within the granulation tissue of 7 day-old wounds.

Serial sections taken at the greatest wound width were stained with hematoxylin, eosin, phloxine and saffron (HEPS) stain. The neo-epidermal coverage, based on the percentage of the total wound width covered by new epithelium (sum of the epithelial tongues migrating from the right and left wound margins), and the remaining wound width (epithelial gap length) were calculated for each section (Fig 1A). Measurements were determined relative to a constant reference (thickness of the intact epidermis adjacent to the wound). Each measurement for each criterion was taken twice and averages were used for statistical analyses. Total wound width was measured on days 7 and 14, whereas neo-epidermal coverage and the remaining wound width were measured on day 7.

The area of granulation tissue was quantified in day 7 wound samples using Zen Pro 2012 software and an Axioimager M1 microscope (Zeiss, Toronto, Ontario, Canada) (Fig 1B). Capillaries within the granulation tissue were counted; only blood vessels running perpendicularly to the wound surface were included in the count (Fig 1C). The average number of capillaries was then divided by the granulation tissue area measurement obtained for that section, to obtain the capillary density.

### Statistical analyses

Statistical testing was done using Prism v6.0d software (GraphPad software, Inc. La Jolla, CA, USA). The threshold of statistical significance for all analyses was defined as *P* ≤ 0.05. The statistical analyses applied to specific data sets are detailed in the corresponding figure legends.

## Results

### Part I: Molecular profile

The wellbeing of all experimental mice was judged to be good, as assessed through the analysis of altered behavioural and facial expressions.

**Fig 2:**
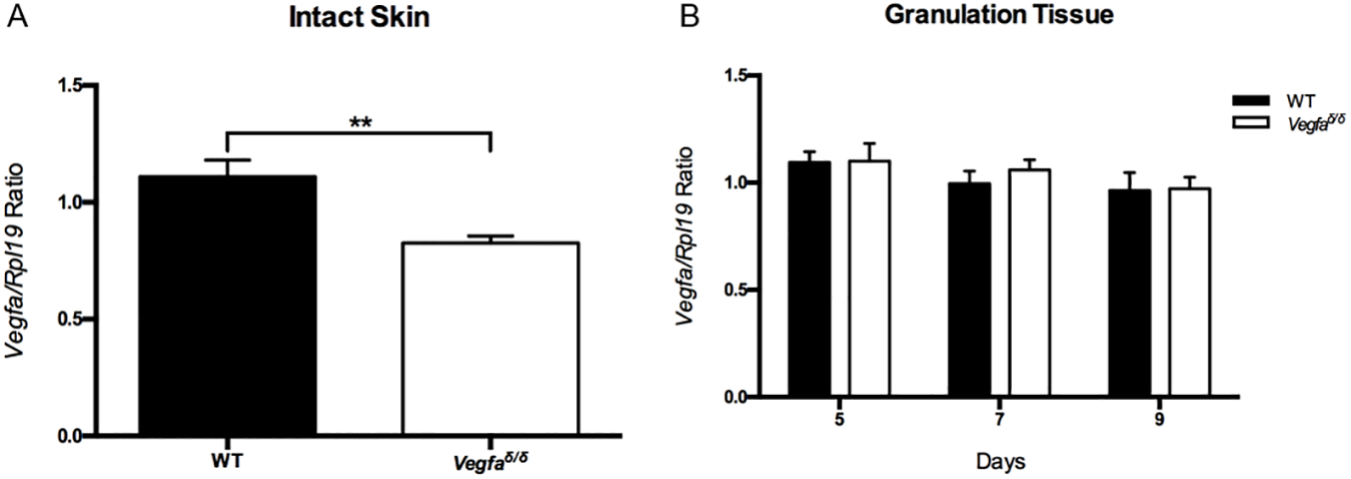
*Vegfa* mRNA levels in intact skin and in granulation tissue in wild type and mutant (*Vegfa*^∂/∂^) mice. (A) *Vegfa* mRNA levels in intact skin. (B) *Vegfa* mRNA levels in granulation tissue at the indicated days post-wounding. Values represent means ± SEM (n = 6). A two-tailed unpaired t-test was used to analyze data from intact skin. A Sidak-corrected repeated measures 2-way ANOVA was used to analyze data from granulation tissue. **: values are significantly different, P = 0.0044. *Vegfa*: Vascular endothelial growth factor-a.

*Vegfa* mRNA levels in wild type and in mutant mice lacking a functional HRE in the *Vegfa* promoter (*Vegfa*^∂/∂^) were determined in both intact skin and granulation tissue throughout the course of wound healing. *Vegfa* mRNA levels in intact skin from *Vegfa*^∂/∂^mice were significantly lower compared to those of their wild type counterparts (Fig 2A) however, unexpectedly, no differences in *Vegfa* mRNA levels in granulation tissue were detected between genotypes during wound healing (Fig 2B).

**Fig 3:**
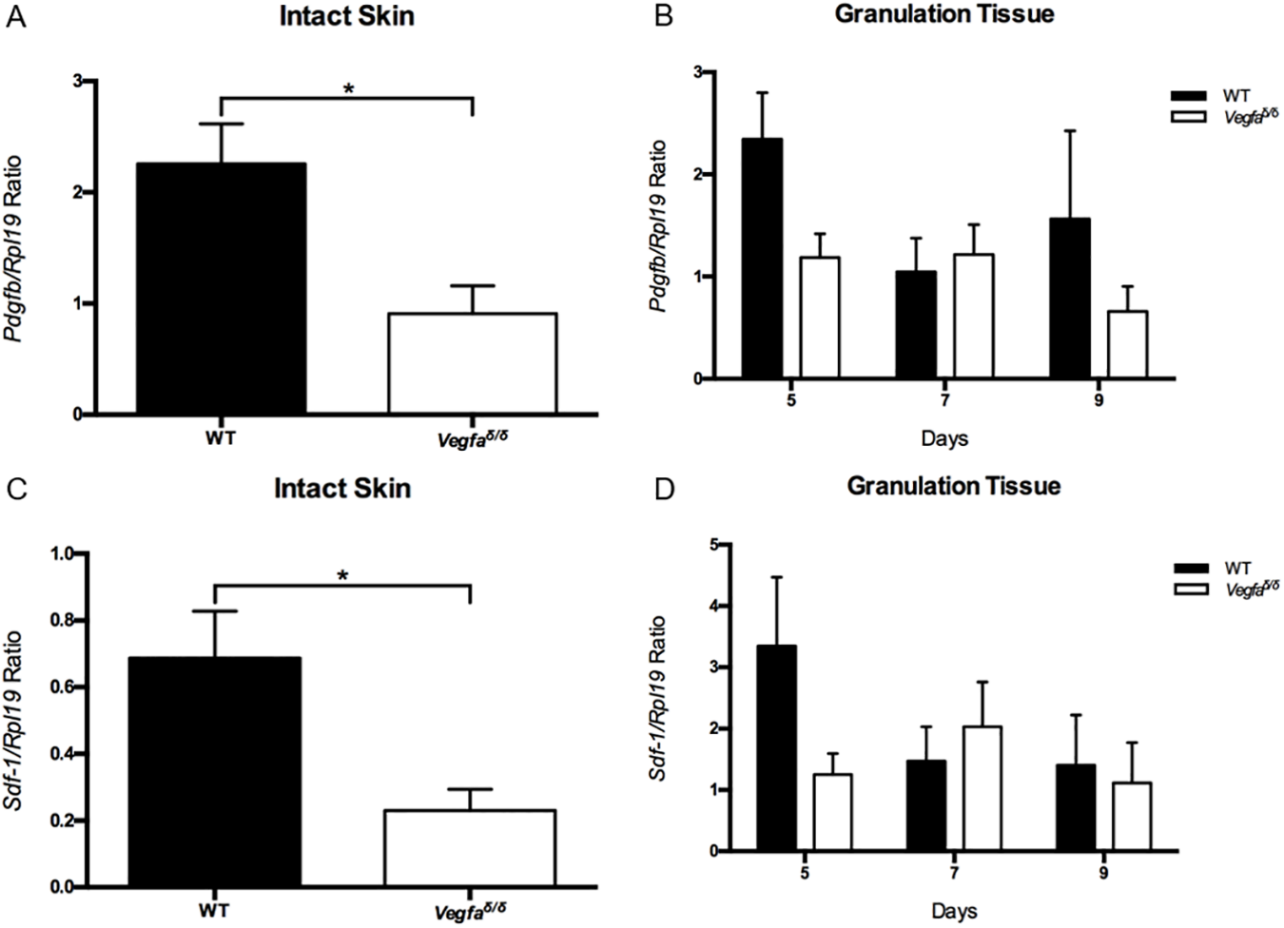
*Pdgfb and Sdf-1* mRNA levels in intact skin and in granulation tissue in wild type and *Vegfa*^∂/∂^ mice. (A) *Pdgfb* mRNA levels in intact skin, *: values are significantly different, P = 0.0119. (B) *Pdgfb* mRNA levels in granulation tissue. (C) *Sdf-1* mRNA levels in intact skin, *: values are significantly different, P = 0.0145. (D) *Sdf-1* mRNA levels in granulation tissue. Messenger RNA levels are relative to that of *Rpl19*. Values represent means ± SEM (n = 6). A two-tailed unpaired t-test was used to analyze data from intact skin. A Sidak-corrected repeated measures 2-way ANOVA was used to analyze data from granulation tissue. *Pdgfb*: Platelet-derived growth factor-b; *Sdf-1*: Stromal-derived factor-1; *Rpl19*: 60S ribosomal protein L19.

Messenger RNA levels of downstream VEGFA angiogenic target genes, *Pdgfb* and *Sdf-1*, were subsequently measured to verify if they reflected levels of *Vegfa*. *Pdgfb* and *Sdf-1* mRNA levels in intact skin mirrored *Vegfa* mRNA levels, being significantly lower in mutant mice than in wild type mice (Figs 2C and 3A). Neither *Pdgfb* nor *Sdf-1* mRNA levels in granulation tissue differed amongst genotypes during the healing process (Fig 3B and 3D).

**Fig 4:**
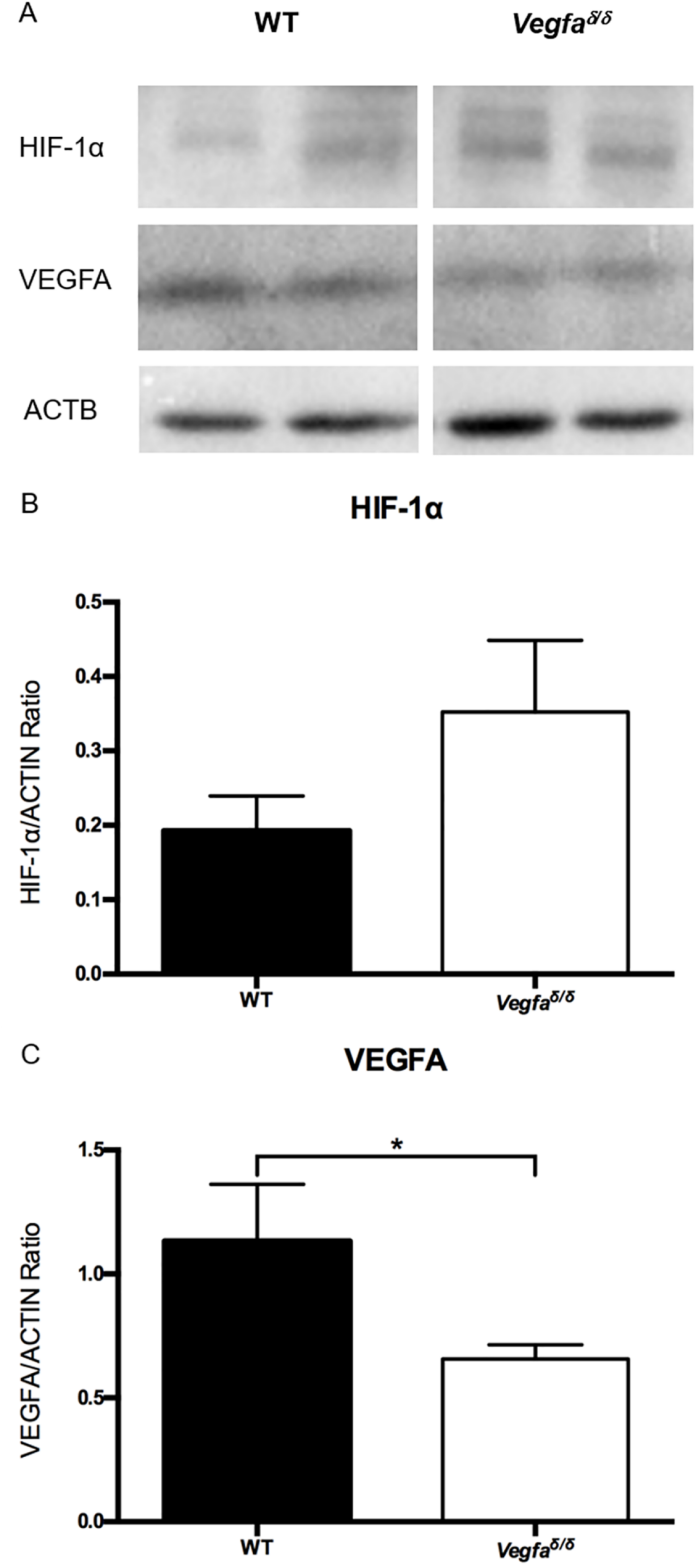
HIF-1α and VEGFA expression in intact skin of wild type and *Vegfa*^∂/∂^ mice. (A) Representative immunoblot analyses of HIF-1α and VEGFA expression in intact skin (n = 2/genotype). (B) Quantification of HIF-1α immunoblots (n = 6/genotype). Data were analyzed using a two-tailed unpaired t-test. (C) Quantification of VEGFA immunoblots (n = 6/genotype). Data were analyzed using a Mann-Whitney test. *: values are significantly different, P = 0.0152.

HIF-1a: Hypoxia-inducible factor 1 alpha; VEGFA: Vascular endothelial growth factor-a.

HIF-1α and VEGFA expression in intact skin were analyzed by immunoblotting (Fig 4A). Whereas HIF-1α expression did not differ between genotypes (Fig 4B), VEGFA expression in intact skin was significantly lower in mutant mice than in wild type mice (Fig 4C).

### Part II: Wound healing assay

**Fig 5:**
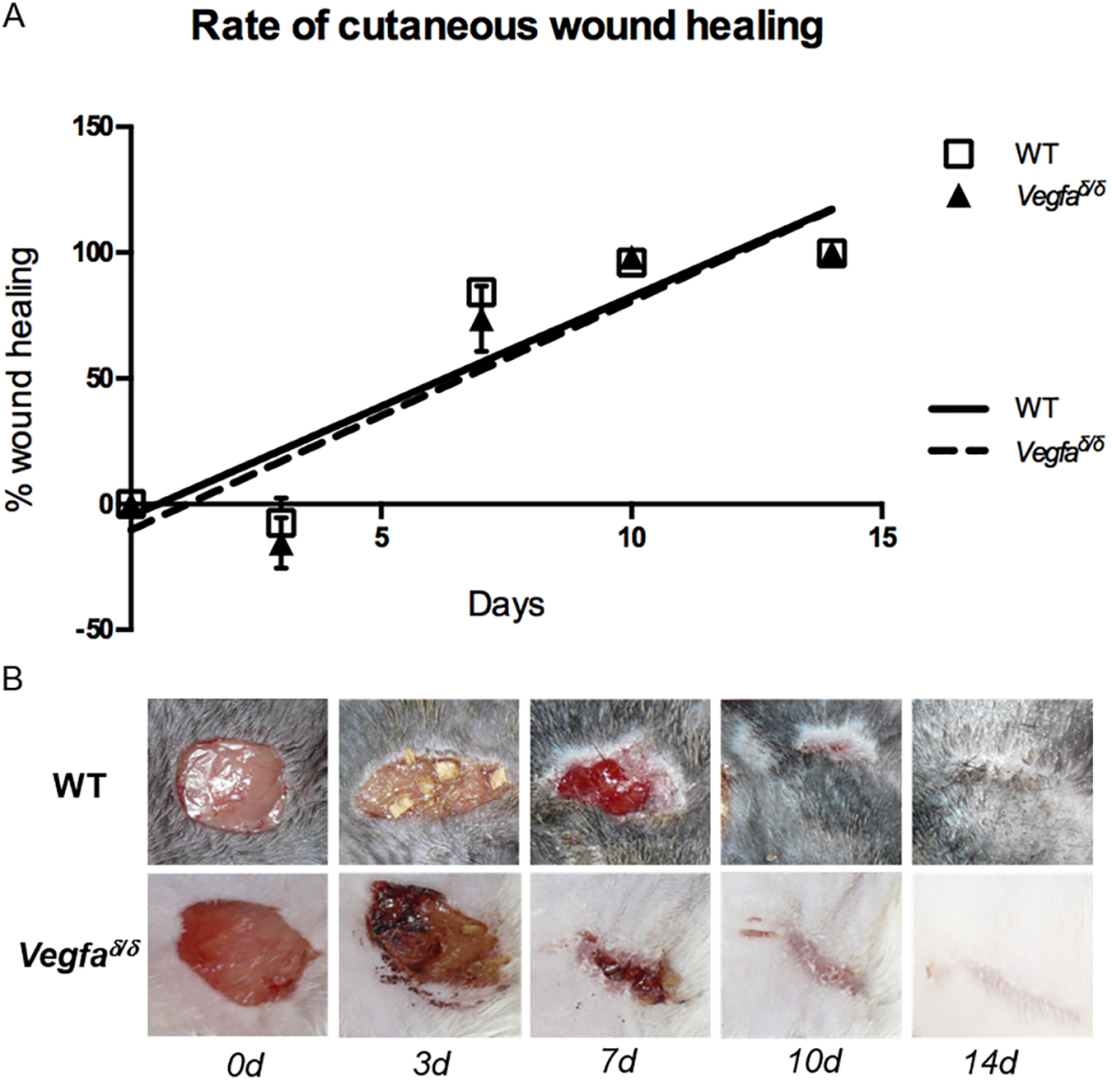
Healing kinetics and macroscopic appearance of wounds in wild type and *Vegfa*^∂/∂^ mice. (A) Percent wound healing over time. Values represent means ± SEM (n = 8 for WT; n = 7 for *Vegfa*^∂/∂^). A Bonferroni-corrected one-way ANOVA was used to analyze the data. (B) Representative photographs of the healing wounds at the indicated days post-wounding (0d = immediately post-operative). WT: wild-type; *Vegfa*: Vascular endothelial growth factor-a.

The overall decrease in wound surface area over time did not differ significantly between wild type and mutant mice (Fig 5A). The majority of wounds had fully healed by day 14 of experimentation (Fig 5B).

**Fig 6:**
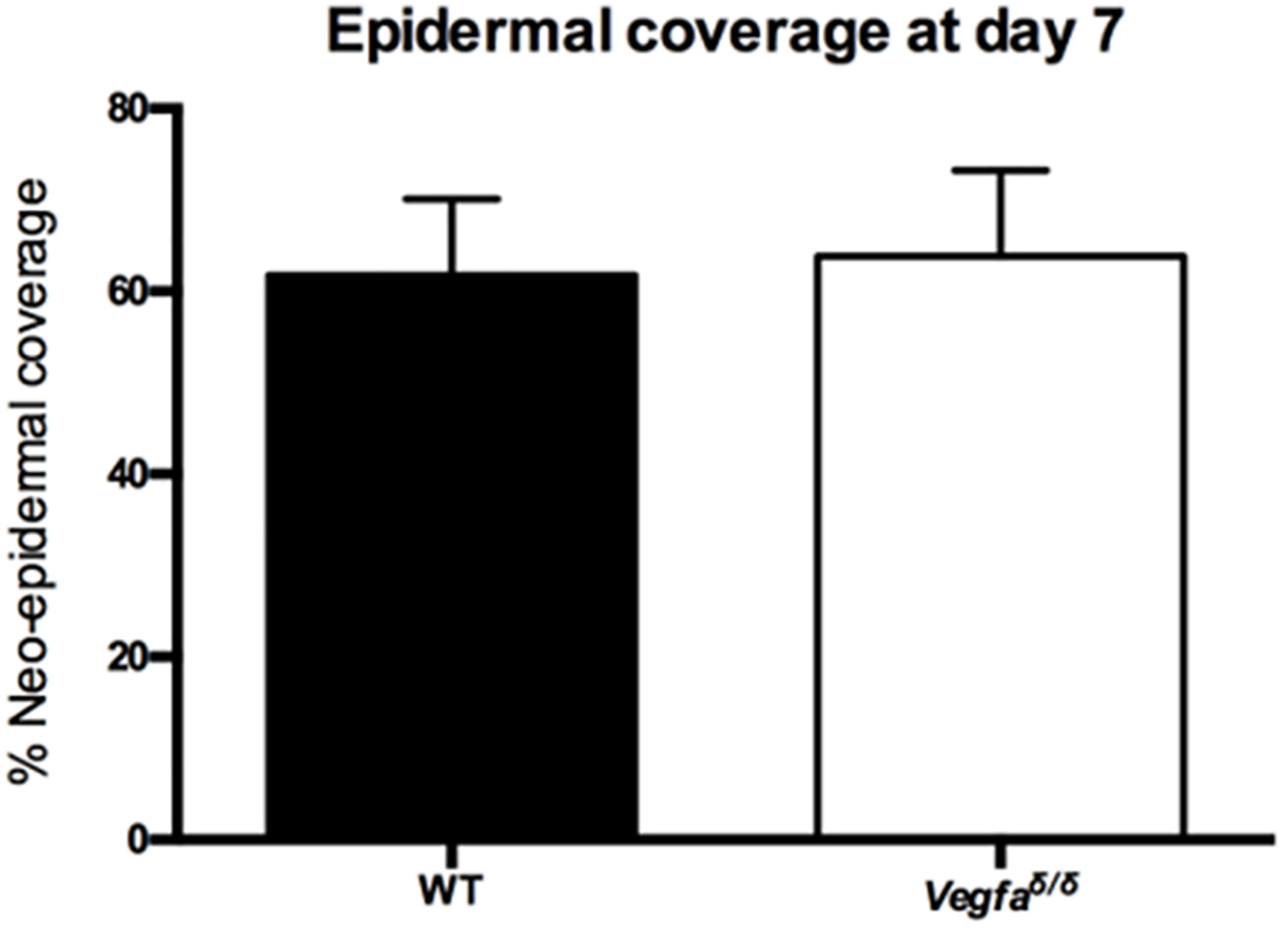
Epithelialization of healing wounds in wild type and *Vegfa*^∂/∂^ mice. Quantitative analysis of neo-epidermal coverage in the healing wounds of WT and *Vegfa*^∂/∂^ mice. Data were analyzed using a two-tailed unpaired t-test. Values represent means ± SEM (n = 8 for WT; n = 6 for *Vegfa*^∂/∂^). WT: wild-type; *Vegfa*: Vascular endothelial growth factor-a.

Since VEGF is known to contribute to keratinocyte migration [21], histometric analyses were done to examine potential epidermal differences in the wounds of wild type and mutant mice. Quantification of neo-epidermal tongue lengths and epithelial gaps showed no difference between the wounds of wild type and mutant mice at day 7 (not shown). Consequently, no significant difference in neo-epidermal coverage was found between the two populations of mice (Fig 6).

**Fig 7:**
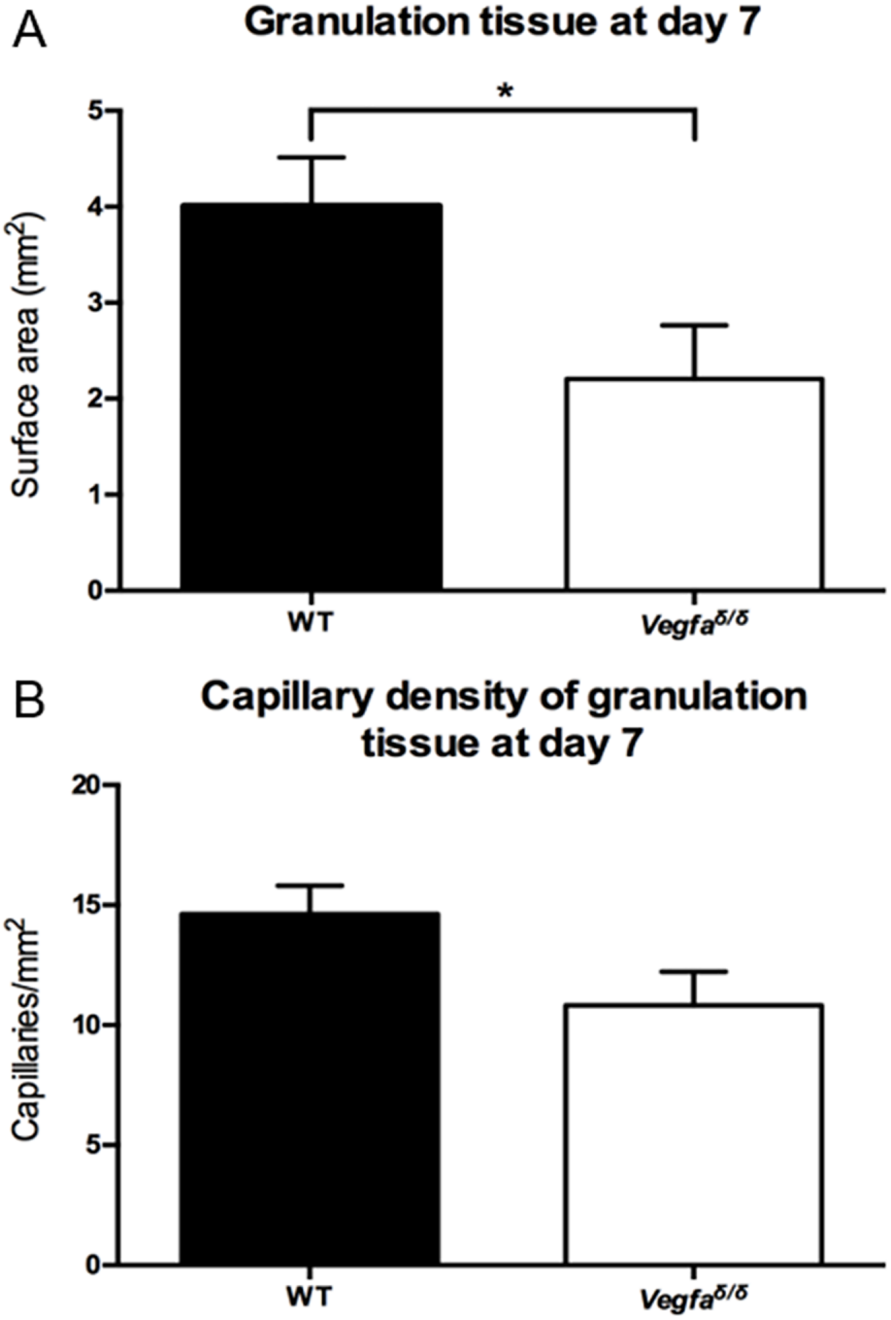
Content of granulation tissue and capillaries in wild type and *Vegfa*^∂/∂^ mice. (A) Surface area of the granulation tissue in 7 day-old wounds, *: values are statistically different, P = 0.0354. Values represent means ± SEM (n = 8 for WT; n = 6 for *Vegfa*^∂/∂^). A two-tailed unpaired t-test was used to analyze the data. (B) Capillary density (Cp / mm^2^ of granulation tissue). Values represent mean ± SEM (n = 8 for WT; n = 6 for *Vegfa*^∂/∂^). A two-tailed unpaired t-test was used to analyze the data. WT: wild-type; *Vegfa*: Vascular endothelial growth factor-a.

Because VEGFA is a major stimulator of angiogenesis in response to the hypoxia generated by wounding, the blood vessel-rich granulation tissue present within 7 day-old wounds was examined. Granulation tissue surface area was measured in cross-sections as an approximation of granulation tissue volume. A significantly smaller surface area of granulation tissue was present in the wounds of mutant mice (Fig 7A). Furthermore, there was a trend towards reduced capillary density within the granulation tissue of 7 day-old wounds in the mutant mice compared to the wild type mice (P = 0.0602) (Fig 7B).

## Discussion

Hypoxia associated with cutaneous wounding triggers the expression of a number of genes required for the healing process, including the key angiogenic factor VEGFA [4, 22]. Although it is established that HIF-1 can regulate *Vegfa* expression in several cell types via a HRE present in the *Vegfa* promoter [7], that this regulatory process drives *Vegfa* expression in the context of cutaneous wound healing has long been presumed, but not actually demonstrated. In the present study, we employed the *Vegfa*^∂/∂^ model, in which the lack of a functional HRE in the *Vegfa* promoter provided the ideal system to determine the contribution of HIF-1 to *Vegfa* expression in wounded skin.

Contrary to our hypothesis, *Vegfa* expression was not compromised in the granulation tissue in the wounds of *Vegfa*^∂/∂^ mice. The simplest explanation for this finding is that HIF-1 is simply not a regulator of *Vegfa* in the context of wound healing. Indeed, many additional factors and signaling processes are known to regulate *Vegfa* expression and may be relevant in granulation tissue. These include epidermal growth factor, platelet-derived growth factor and interleukin-1alpha, which are released in response to wounding [23]. Physicochemical stimuli such as acidosis, hypoglycemia, reactive oxygen species and mechanical stretching all stimulate *Vegfa* expression [2, 3, 13, 24, 25] and occur within the wound environment. Likewise, the extracellular signal-regulated kinase (ERK)1/ERK2 pathway [26, 27] induces *Vegfa* expression through the binding of transcriptional factor complexes such as adaptor complex 2 [26, 28–30] and specificity protein 1 [26, 28–30] to the *Vegfa* promoter [26]. Whether these or other signaling processes drive *Vegfa* expression in granulation tissue will be grounds for further study. Another explanation for unaltered *Vegfa* expression in the granulation tissue of *Vegfa*^∂/∂^ mice is that compensatory mechanisms (perhaps including the aforementioned *Vegfa* regulatory processes) may have been induced to augment *Vegfa* expression, thereby masking any deficit caused by deficient HIF-inducibility of the *Vegfa* promoter. Further experiments will be required to test this possibility.

In this study, we readily detected HIF-1alpha protein in intact skin, confirming previous reports of constitutive expression in several functionally diverse cell types [31–34] including those populating skin [35]. The decreased levels of *Vegfa* expression in the skin of *Vegfa*^∂/∂^ mice therefore indicate that one role of the constitutively expressed HIF-1α is to ensure proper basal expression of *Vegfa*.

Despite normal expression of *Vegfa* (and *Pdgfb* and *Sdf1*) in their granulation tissue, wounds in *Vegfa*^∂/∂^ mice featured a reduced abundance of granulation tissue, and capillary density was decreased within the granulation tissue. Although we cannot provide a definitive explanation for this, deficient *Vegfa* expression in the intact skin of *Vegfa*^∂/∂^ mice (i.e., prior to wounding) provides a plausible explanation. Upon wounding, decreased VEGFA levels present locally within the skin may have led to delayed and/or reduced capillary and granulation tissue formation in the early phases of healing, leading to the changes observed 7 days post-wounding in the *Vegfa*^∂/∂^ model. This would suggest that a certain level of VEGFA expression in intact skin is required for an optimal response to wounding and a rapid onset of the healing process. Surprisingly, in spite of defects in the granulation tissue, the wounds of *Vegfa*^∂/∂^ mice healed at the same rate as the wounds of wild type mice. This might be explained by the highly contractile nature of wound healing on the backs of mice, due to the presence of the *panniculus carnosus* muscle in the skin surrounding the wound [36]. Indeed, several studies of cutaneous wound healing in mice employ stents/splints to prevent wound contraction [37, 38], thereby permitting a better analysis of the epithelialization process. Future studies of the *Vegfa*^∂/∂^ mice could employ stenting in order to more conclusively determine if the granulation tissue defects specifically affected epithelialization.

In summary, HIF-1 is not a major regulator of *Vegfa* expression during wound healing; rather, it serves to maintain basal levels of expression of *Vegfa* and its target genes in intact skin, which are required for optimal granulation tissue formation in response to wounding.
